# Physical Activity, Social Support, and Mood in Older Adults during the COVID-19 Pandemic

**DOI:** 10.1093/geroni/igab046.2692

**Published:** 2021-12-17

**Authors:** Morgan Inman, Clare Murphy, Jessica Strong

**Affiliations:** 1 University of Prince Edward Island, University of Prince Edward Island, Prince Edward Island, Canada; 2 University of Prince Edward Island, Charlottetown, Prince Edward Island, Canada

## Abstract

Research shows that increased physical activity is associated with improved mood and reduced symptoms of depression in older adults. Prior research has also found that loneliness and social isolation have a significant impact on the mental and physical well-being of older adults, with higher amounts of social connectedness and social activity associated with more frequent positive mood states. Overall social isolation is increased due to the COVID-19 pandemic and this could have a large impact on the physical and mental health of older adults. A group of 36 community dwelling older adults (Mean age = 70.5) completed questionnaires measuring physical activity, social activity, and social support, during the COVID-19 pandemic. Analyses found that perceived social support and average social network size significantly predicted positive mood states (F(2,33)=3.32, p<0.05) accounting for 16.7% of the variance, with a large effect. After adding average number of hours of sedentary activity the model was not significant. Perceived social support was more predictive of positive mood (β=0.32) compared to network size (β=0.17). There was a trend for the same three variables to predict negative mood (F(3,32)=2.76, p=0.06) accounting for 22% of the variance. Sedentary behaviour was the most predictive (t=2.68, p<0.05, β= 0.49). This suggests that perceived social support is most predictive of positive mood, and sedentary behaviour is predictive of negative mood during the COVID-19 pandemic.

